# Therapeutic Effects of Iranian Traditional Medicine on a Patient With Cryptogenic Cirrhosis

**DOI:** 10.5812/ircmj.16548

**Published:** 2014-07-05

**Authors:** Hamid Shamsi Baghbanan, Esmaeil Nazem, Saeideh Yarjoo, Bagher Minaei

**Affiliations:** 1 School of Traditional Medicine, Shahid Beheshti University of Medical Sciences, Tehran, IR Iran; 2 School of Traditional Medicine, Tehran University of Medical Sciences, Tehran, IR Iran

**Keywords:** Cryptogenic Cirrhosis, Iranian Traditional Medicine, Herbal

## Abstract

**Introduction:**

There are several disorders that cause hepatic cirrhosis. However, if there is no known cause for cirrhosis, it is called cryptogenic cirrhosis. Cirrhosis is believed to be irreversible in its late stages. In these cases, liver transplantation is the only solution.

**Case Presentation:**

The study case was a 29-year-old man, admitted to the hospital four years ago due to esophageal variceal hemorrhage. A biopsy of his liver showed cryptogenic cirrhosis; thus, he was a candidate for liver transplant. The patient visited the outpatient Iranian traditional medicine center, Behesht Clinic, in Tehran, Iran, two years after his bleeding course and began treatment with traditional herbal medicine. In the following month, he stopped taking his previous medications. During the 18-month follow up, he was visited 16 times. During this time, his general health improved and his hemoglobin level increased. Based on the ultrasound reports, the spleen size, the gallbladder wall edema, and the portal vein diameter decreased. Even though the ascites disappeared, the patient gained weight. His model for end-stage liver disease (MELD) score reduced from 10 (prior to the Iranian traditional medicine treatment) to 8. The bilirubin level decreased as well. The alanine aminotransferase (ALT) and aspartate aminotransferase (AST) levels increased and the serum albumin level and platelet count decreased.

**Conclusions:**

In this patient, traditional medicine treatment improved the patient's general well-being, hematopoiesis and portal hypertension. Furthermore, it improved his quality of life, although it had no effect on his liver function. We recommend more clinical trials on therapeutic effects of Iranian traditional medicine on cryptogenic cirrhosis.

## 1. Introduction

Cirrhosis can be described as advanced stage of progressive hepatic fibrosis. Some of the known causes for cirrhosis are chronic viral hepatitis and alcoholism. If the source is unknown, it is referred to as cryptogenic cirrhosis (1). Sometimes cirrhosis can be treated in early stages by removing the concussive substance (like alcohol); but, the ultimate treatment for decompensated cryptogenic cirrhosis is liver transplantation (2). Nine percent of all liver transplantations are due to cryptogenic cirrhosis (3). In this group, chronic rejection rate and postoperative mortality rate are higher, and survival rates at 5, 10, and 15 years are lower, compared to other etiologies (4). The end stage liver disease manifests with symptoms such as variceal hemorrhage, ascites, and encephalopathy, and if decompensation happens, survival becomes considerably impaired. If possible, it is reasonable to treat decompensated cryptogenic cirrhosis with preparations that can reverse or slow down its progression.

A patient with decompensated cryptogenic cirrhosis was admitted to hospital due to esophageal variceal hemorrhage and was eventually considered for liver transplantation. He was treated with Iranian traditional medicine for 18 months.

## 2. Case Presentation

The patient was a 29-year-old man admitted to the hospital four years ago (June 2009) due to hematemesis. The problem was diagnosed as esophageal variceal bleeding and the proper treatment was provided. He had no history of alcohol consumption or diabetes mellitus. Moreover, the tests were negative for all types of viral hepatitis (B, C), EBV (Epstein–Barr virus), herpes, CMV (Cytomegalovirus), autoimmune hepatitis, HIV, celiac and Wilson’s disease. The colonoscopy result was normal. On April 2010, liver biopsy showed cirrhotic changes and the patient was diagnosed with cryptogenic cirrhosis. His name went to the list of liver transplantation candidates and the academic management for cirrhotic patients was started for him.

The patient first visited Behesht Clinic of Tehran University of Medical Sciences in Tehran for Iranian traditional medicine on September 2011, about 17 months after being diagnosed. At the time, his medicinal prescription included spironolactone, propranolol, prednisolone and doxepin. The patient stopped taking all the medications after one month.

His height was 173 cm and his weight was 57 kg. In his first visit, he had flatulence, dyspepsia, and heartburn. He was generally thirsty and drank up to eight glasses of cold water a day. He also had severe itching sensation of skin and would not sweat even during intense physical activities. His sclera was icteric.

From his first visit to Behesht Clinic on September 2011 till February 2013, the patient was visited 16 times and each time, considering his general state and by performing physical examinations, the necessary traditional medication was prescribed for him. After three weeks of treatment, his itching sensation was significantly reduced, he felt energetic, and his flatulence and heartburn decreased. During four months of treatment, the patient gained 6 kg without any sign of ascites in abdominal ultrasonography. From the first admission (June 2009) until the end of study (February 2013), the alpha-fetoprotein (AFP) level was always in the normal range. The traditional medicine preparations used for this patient were based on the book “Al-Qanoon fi al-Tibb” by Avicenna. What follows is a list of different medicines used at different stages of the treatment:

Monzeje soda, kabed capsuls, sekanjebine-bozoori, sekanjebine-sadri, samgh capsuls, eksir syrup, khabasolhadid, goleghand, habolroman, javareshe amole, aftimoon syrup, araghe-kasni shahtare, araghe-zenyan. Tables 1 and 2 show the changes in the patient’s test results before and after the traditional medication. At the moment, the patient is in a good general condition and there is no need for liver transplantation.

**Table 1.** Results of the Ultrasound and Biopsy Before and After the Traditional Medication ^a^

Date and Method of Diagnosis	Liver	Portal Vein	Gallbladder Wall Thickness, mm	Spleen parameters, mm	Abdomen
**June 2009, ultrasound**	Normal size, heterogenic echo	Diameter: 12.5 cm, blood stream velocity: 13 cm/s	4	Size: 70 × 175, spleen vein diameter: 11.4	
**March 2010, ERCP**	Hepatic cirrhosis	Portal hypertension			
**April 2010, liver biopsy**	chronic hepatitis, modified HAI grade: 5-6/18 , stage: at least 4-5/6				
**August 2010**	Heterogenic echo	Portal vein diameter outside liver: 13.4, blood stream velocity: 10 cm/s, portal vein diameter inside liver: 11.7 cm	6.5	size: 120 × 190, homogenic echo, spleen vein diameter: 11	A little ascites in the pelvic area
**January 2011, ultrasound** ^******b**^	Heterogenic echo	Portal vein diameter outside liver: 13.2, blood stream velocity: 7.5 cm/s, portal vein diameter inside liver: 11 mm	5	size: 77 × 195, spleen vein diameter: 10, blood stream velocity: 22 cm/s	No free fluid in the abdomen or pelvis
**May 2012, ultrasound**	Heterogenic echo	Portal vein diameter: 12.7 mm, the hepatic vein diameter was normal	5	spleen size: 81 × 180	No free fluid in the abdomen or pelvis

^a^ Abbreviations: ERCP, endoscopic retrograde cholangiopancreatography; HAI, hepatic activity index.

^b^ After this date Iranian Traditional Medicine was used.

**Table 2.** The Laboratory Tests Results Before and After the Traditional Medication ^a^

Dates	WBC x 10^3^/µL	Hb g/dL	HCT%	RBC × 10^6^/µL	Plat × 1000/mL	AST U/L	ALT U/L	ALK P U/L	Albumin g/dL	Bili Total mg/dL	Bili Direct mg/dL	PT	PT patient activity %	INR	PTT Sec	BUN mg/dL	Cr mg/dL	Total Protein g/dL	Albumin g/dL	Globulin g/dL	A/G	Iron mcg/dL	Tibc mcg/dL	Ferritin ng/mL
**2009/7/11**	6300	10.2	32.7	3.74	128	97	34	582		1.6	0.4	12.7	79	1.16		11	0.9	6	3.7	2.3	1.6	56	353	50
**2010/2/28**	3700	10.2	30.6	5.17	102	101	55	569		1.9	1.2	12.3	100	1		13	0.84	7.9	4.4	3.5	1.2			
**2010/4/20**	4400	7.6	26.4	4	129	101	66	785		1.4	0.7	13			25	31	0.8							
**2010/7/7**	2900	8.4	28.2	4.73	88	62	34	284	4.3	1.2	0.4	14	69	1.24		13	0.93							
**2010/11/23**	3000	6.4	20.5	3.84	117	153	70	595				12.9	89	1.07		20	0.81	6.8	4.2	2.6	1.6			
**2011/1/8**		7	26.9	3.79	129	73	89	632	4.5	1.2	0.4	13		1										
**2011/9/4**	4000	8.3	31.6	4.4	85	57	24	804		4.61	2.98	12	100	1		10	0.7	7.4	3.8			62	477	5.43
**2011/11/17** ^**b**^	2200	8.3	28.3	4.02	92	56	31	941		0.6	0.3	15.5		1.3	41	8.3	0.7	7.4	3.9			63	459	3.4
**2012/2/20**	2100	7.6	29	4.15	58	118	65	1134		1.3	0.4					9.8	0.9					45	390	4.42
**2012/5/19**	2200	8.9	33.1	4.79	58	45	74	999		1.3	0.4	13	100	1	35	13	0.7	7.5	4.7			35	331	3.31
**2012/8/21**	3300	10.3	36.7	5.15	149	77	55		4	1.1	0.5	13		1		14	1.1	7.2				54	397	8.63
**2012/11/1**	2300	13.1	41	4.5	54	119	61		3.1	1.2	0.6	13.8	90.3	1.1		10	0.5	6.5				60	318	20.7

^a^ Abbreviations: ALKP, alkaline phosphatase; ALT, alanine transaminase; AST, aspartate aminotransferase; BUN, bloo urin nitrogen; Hb, hemoglobin; HCT, hematocrit; INR, international normalized ratio; PT, prothrombin time; PTT, partial thromboplastin time; Tibc, total iron binding capacity.

^b^ After this, date Iranian Traditional Medication was used.

## 3. Discussion

Hepatic fibrosis causes portal hypertension, which in turn causes esophageal varices. A patient who enters the esophageal variceal hemorrhage phase is actually in decompensation stage and eventually will need liver transplantation. Data presented in Table 1 revealed that after the first admission to the hospital and starting the necessary treatments, the diameter of portal vein increased, but after the beginning of traditional medication it decreased. The spleen size and the gallbladder wall edema also reduced after the treatment and the ascites disappeared. The increase of portal vein diameter, spleen size, gallbladder wall edema, and ascites were resulted from hepatic fibrosis and cirrhosis (5) and after the traditional medication all these symptoms were improved. Therefore, traditional medicine played a positive role in controlling the symptoms and complications of cirrhosis. In some studies, a number of herbal medicines were somehow successful in reversing cirrhosis in animal models (6, 7). Furthermore, positive effects of some herbal medicines in treating cirrhosis in humans have been reported (2, 8).

After treating with traditional medicine, positive changes in hemoglobin, hematocrit, ferritin, Total iron binding capacity (TIBC), and serum iron levels were observed, which were good signs of improvement in patient’s hematopoiesis. These results were expected in traditional medicine, because in this approach, liver is responsible for hematopoiesis and when liver condition improves, hematopoiesis function enhances. Moreover, improvements in symptoms like itching, thirst, digestive signs, general health, and gaining weight without ascites, are signs of a good functioning liver according to traditional medicine. On the other hand, the patient’s platelet count prior to treatment was 85000/mL, and by the end of the study, it was reduced to 54000/mL; replacing prednisolone with traditional medications did not stop the decrease of platelet number in the patient.

Aspartate transaminase (AST) and alanine transaminase (ALT) levels decreased after the beginning of treatment with classical medicine; but with the traditional medicine replacing the previous drugs, AST, ALT and alkaline phosphatase (ALKP) levels started to increase again. The patient’s serum albumin and total protein levels also decreased after the traditional medication. These changes meant that the classical medicine may have better effects on liver function than traditional medicine. Several preparations were used in treating this patient and each of them were made from different herbs based on the book “Al-Qanoon fi al-Tibb”. Effects and other information of these drugs are available (9, 10). Since this article aimed at showing the treatment effects of Iranian traditional medicine, not focusing on the structure and effect of a specific drug, and also due to study limitations, these drugs were not described in details.

Although in this case Iranian traditional medication did not have a positive effect on liver function, it had positive effects on quality of life, hematopoiesis, and portal hypertension. Therefore, based on the above experience, we recommend more studies on therapeutic effects of Iranian traditional medicine on cryptogenic cirrhosis, in form of clinical trials.
